# Supplementary material for: Phenotypic Investigation and RNA-seq of KN1 Involved in Leaf Angle Formation in Maize (Zea mays L.)
Source: Int J Mol Sci. 2024 Mar 10;25(6):3180. doi: 10.3390/ijms25063180 (PMC10970149; doi:10.3390/ijms25063180)
Supplement: Supplementary file 1 [file ijms-25-03180-s001.zip › Figure S1.pdf]

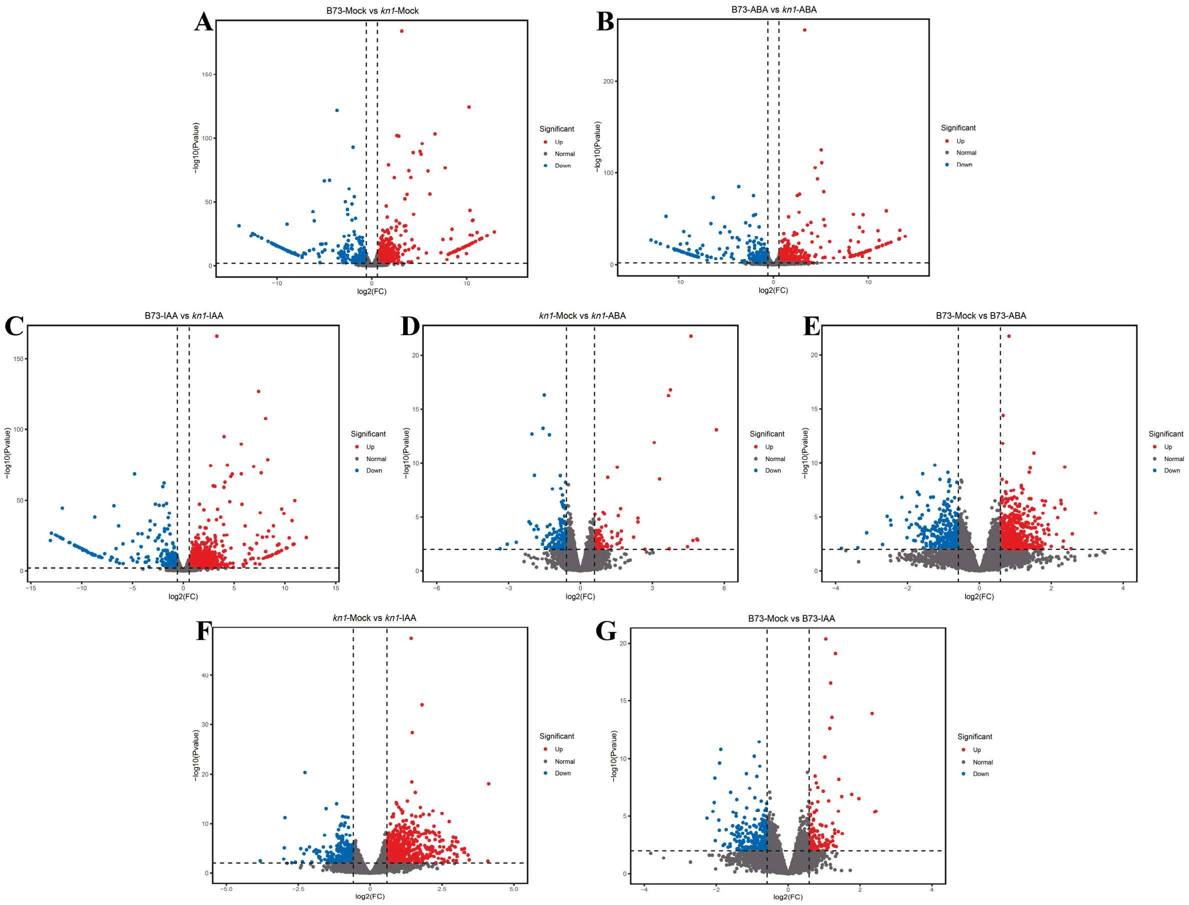

Figure S1: Statistical analysis of DEGs produced by B73 and kn1 under different experimental conditions. (A) Volcano plot of DEGs identified in the comparisons of B73-Mock vs kn1-Mock; (B) Volcano plot of DEGs identified in the comparisons of B73-ABA vs kn1-ABA; (C) Volcano plot of DEGs identified in the comparisons of B73-IAA vs kn1-IAA; (D) Volcano plot of DEGs identified in the comparisons of kn1-Mock vs kn1-ABA; (E) Volcano plot of DEGs identified in the comparisons of B73-Mock vs B73-ABA; (F) Volcano plot of DEGs identified in the comparisons of kn1-Mock vs kn1-IAA; (G) Volcano plot of DEGs identified in the comparisons of B73-Mock vs B73-IAA. Note: Mock represents exogenous ABA or IAA concentration of 0 μM, namely the control group. Red, blue and gray dots in the volcano plot represent up-regulated, down-regulated and non-differentially expressed genes, respectively.
